# The LongitudinAl Nationwide stuDy on Management And Real‐world outComes of diabetes in India over 3 years (LANDMARC trial)

**DOI:** 10.1002/edm2.422

**Published:** 2023-07-01

**Authors:** Ashok K. Das, Sanjay Kalra, Shashank Joshi, Ambrish Mithal, K. M. Prasanna Kumar, A. G. Unnikrishnan, Hemant Thacker, Bipin Sethi, Subhankar Chowdhury, Amarnath Sugumaran, Ashwini Satpathy, Arvind Gadekar, Shalini K. Menon, Renuka Neogi, Deepa Chodankar, Chirag Trivedi, S. K. Wangnoo, A. H. Zargar, Nadeem Rais, LANDMARC study group

**Affiliations:** ^1^ Mahatma Gandhi Medical College and Research Institute Sri Balaji Vidyapeet Puducherry India; ^2^ Bharti Hospital Karnal India; ^3^ Lilavati Hospital Mumbai India; ^4^ Medanta‐ The Medicity Gurgaon India; ^5^ Centre for Diabetes and Endocrine Care Bengaluru India; ^6^ Chellaram Diabetes Institute Pune India; ^7^ Bhatia Hospital Mumbai India; ^8^ Care Hospital Hyderabad India; ^9^ IPGME and R and SSKM Hospital Kolkata India; ^10^ Sanofi Mumbai India; ^11^ Apollo Hospital Education and Research Foundation New Delhi India; ^12^ Center for Diabetes & Endocrine Care Srinagar India; ^13^ Chowpatti Medical Centre Mumbai India

**Keywords:** diabetes‐related complications, glycemic control, India, LANDMARC, real‐world evidence, T2DM, treatment pattern

## Abstract

**Introduction:**

LANDMARC (CTRI/2017/05/008452), a prospective, observational real‐world study, evaluated the occurrence of diabetes complications, glycemic control and treatment patterns in people with type 2 diabetes mellitus (T2DM) from pan‐India regions over a period of 3 years.

**Methods:**

Participants with T2DM (≥25 to ≤60 years old at diagnosis, diabetes duration ≥2 years at the time of enrollment, with/without glycemic control and on ≥2 antidiabetic therapies) were included. The proportion of participants with macrovascular and microvascular complications, glycemic control and time to treatment adaptation over 36 months were assessed.

**Results:**

Of the 6234 participants enrolled, 5273 completed 3 years follow‐up. At the end of 3‐years, 205 (3.3%) and 1121 (18.0%) participants reported macrovascular and microvascular complications, respectively. Nonfatal myocardial infarction (40.0%) and neuropathy (82.0%) were the most common complications. At baseline and 3‐years, 25.1% (1119/4466) and 36.6% (1356/3700) of participants had HbA1c <7%, respectively. At 3‐years, population with macrovascular and microvascular complications had higher proportion of participants with uncontrolled glycemia (78.2% [79/101] and 70.3% [463/659], respectively) than those without complications (61.6% [1839/2985]). Over 3‐years, majority (67.7%–73.9%) of the participants were taking only OADs (biguanides [92.2%], sulfonylureas [77.2%] and DPP‐IV inhibitors [62.4%]). Addition of insulin was preferred in participants who were only on OADs at baseline, and insulin use gradually increased from 25.5% to 36.7% at the end of 3 years.

**Conclusion:**

These 3‐year trends highlight the burden of uncontrolled glycemia and cumulative diabetes‐related complications, emphasizing the importance of optimizing diabetes management in India.

## Abstract 2

The 3‐year data from the longitudinal LANDMARC study provide valuable insights on diabetes‐related complications, disease progression and treatment strategies among people with diabetes in India. In this pan‐India study, at the end of 3‐years, 205 (3.29%) and 1121 (18.02%) participants reported macrovascular and microvascular complications, respectively, with nonfatal myocardial infarction (40.0%; *n* = 82) and neuropathy (81.98%; *n* = 919) being the most common complications. Despite a gradual improvement in glycemic levels, the burden of uncontrolled glycemia (HbA1c > 7%) was considerable (63.4%; *n* = 2344). The LANDMARC study emphasizes the need for optimizing disease management and timely intensification of therapy among people with diabetes in India.

## 1 INTRODUCTION

The constantly rising burden of type 2 diabetes mellitus (T2DM) in India has had a catastrophic impact on the healthcare system primarily due to the development of diabetes‐associated complications and subsequent morbidity and mortality. The chronic nature of the disease course is the key attributable factor for the rising incidence of diabetes‐related complications in this patient population.^1^ Diagnosis of diabetes at an early stage of the disease course is low in India, and more than 50% of diabetes cases are undiagnosed across the countries from South‐East Asia region.^2^ Furthermore, poor glycemic control and subsequent development of complications emphasize the need for optimal diabetes management strategies in India.^3^, ^4^, ^5^

For comprehensive understanding of the Indian scenario, there is a need for real‐world evidence in terms of longitudinal data regarding the glycemic control achieved, the occurrence of diabetes‐related complications during disease progression, and treatment strategies implemented in the Indian diabetic population. The LongitudinAl Nationwide stuDy on Management And Real‐world outComes of diabetes in India (LANDMARC) initiated in 2017 was a novel national, prospective, observational real‐world study carried out in people with T2DM from pan‐India regions to determine the occurrence of diabetes complications, glycemic control and treatment patterns in the routine clinical practice over a period of 3 years. Trends observed in the interim analysis of baseline, 1‐year and 2‐year data have previously been published,^6^, ^7^, ^8^, ^9^, ^10^, ^11^, ^12^ and this article will explore comprehensive trends observed over a 3‐year period.

## 2 METHODS

Details of the design and methodology of the LANDMARC study were published earlier^13^ and are briefly summarized here.

### 2.1 Study design and ethics

The LANDMARC study (Trial Registration No (CTRI/2017/05/008452) was a prospective, multicenter, observational real‐world study investigating a large cohort of people with T2DM across pan‐India sites over a period of 3 years (April 2017–July 2021).

The trial protocol was approved by the relevant Ethics Committee or institutional review board at the study sites. The protocol complies with the Declaration of Helsinki and all subsequent amendments. All participants provided signed informed consent before study participation.

### 2.2 Study participants

This study included participants of either sex, aged 25–60 years at the time of diagnosis of T2DM, with T2DM for at least 2 years at the time of enrolment, with or without glycemic control and on two or more antihyperglycemic therapies. Other details of inclusion and exclusion criteria were summarized in our previous publications.^13^

### 2.3 Data collection

Information related to study end‐points was collected prospectively every six months up to the end of the study at 36 months. The study design was planned to mirror the real‐life management of participants with T2DM; therefore, no assessments were mandated, and the available data were recorded in electronic case report forms (eCRFs). Data quality control was performed by qualified designated personnel. Any adverse drug reaction related to any Sanofi product (clinical signs, laboratory values or other) was reported and followed up until the clinical recovery was complete and laboratory results (if clinically significant) had returned to normal or until progression had been stabilized.

### 2.4 Study end‐points

Study end‐points assessed were subdivided into three subsections: complications, glycemic control and management.

- Complication end‐points were

- proportion of participants with macrovascular disease over 36 months (cumulative incidence of cardiovascular outcomes [nonfatal myocardial infarction {MI}, nonfatal stroke and cardiovascular death] and peripheral vascular disease [PVD]);
- proportion of participants with composite of nonfatal MI/nonfatal stroke/hospitalization for unstable angina/cardiovascular death over 36 months (cumulative incidence);
- proportion of participants with other cardiovascular events such as hospitalization due to acute coronary syndrome (ACS), urgent revascularization procedures, hospitalization for heart failure (HF) or unstable angina at the end of 6, 12, 24 and 36 months;
- development of microvascular complications at 12, 24 and 36 months (neuropathy, nephropathy and retinopathy [initiation of retinal photocoagulation, vitreous haemorrhage and diabetes‐related blindness]);
- composite renal and retinal microvascular outcome that included the first occurrence of any of the following (initiation of retinal photocoagulation, vitreous haemorrhage, diabetes‐related blindness, incident or worsening nephropathy).

B Glycemic control end‐points assessed were

- proportion of participants controlled (HbA1c <7% and <6.5%) at the end of 6, 12, 24 and 36 months;
- proportion of participants controlled (HbA1c <7%)/uncontrolled (HbA1c ≥ 7%) at 12, 24 and 36 months, and the occurrence of microvascular and macrovascular complications in these two groups at the end of 36 months;
- comparison of change in glycemic assessments at 36 months between participants with and without complications at baseline;
- comparison of the glycemic control and outcomes at 36 months in participants with and without complications at baseline.

C Management end‐points were to assess time to treatment adaptation from baseline to 12, 24 and 36 months (any dose change/addition/deletion/drug intensification/titration for OADs, insulin or other injectable antidiabetic agents).

### 2.5 Sample size calculation

The minimum sample size required for this study was 4387 with a two‐sided 99% confidence interval, assuming that the percentage of participants with the composite incidence of nonfatal MI, stroke and cardiovascular death after 3 years would be 3%. Hence, the inclusion of approximately 6300 participants would allow estimating this percentage with a precision of at least 1%, after considering that approximately 30% of the participants will drop out from the study before the end of the third year.^13^

### 2.6 Statistical analysis

Data analyses were done using SAS version 9.4 or higher. The normality of data was assessed using Kolmogorov–Smirnov test. A descriptive analysis was used to present categorical data as numbers and percentages and numerical data as mean and standard deviation (for normally distributed data sets) or median and range (for skewed data sets). Comparative analysis between two groups was performed using an independent sample *t*‐test (continuous variables). McNemar's test was used for comparative analysis between paired sample data. A *p*‐value <.05 was considered statistically significant.

## 3 RESULTS

### 3.1 Demographics and baseline characteristics

**FIGURE 1:**
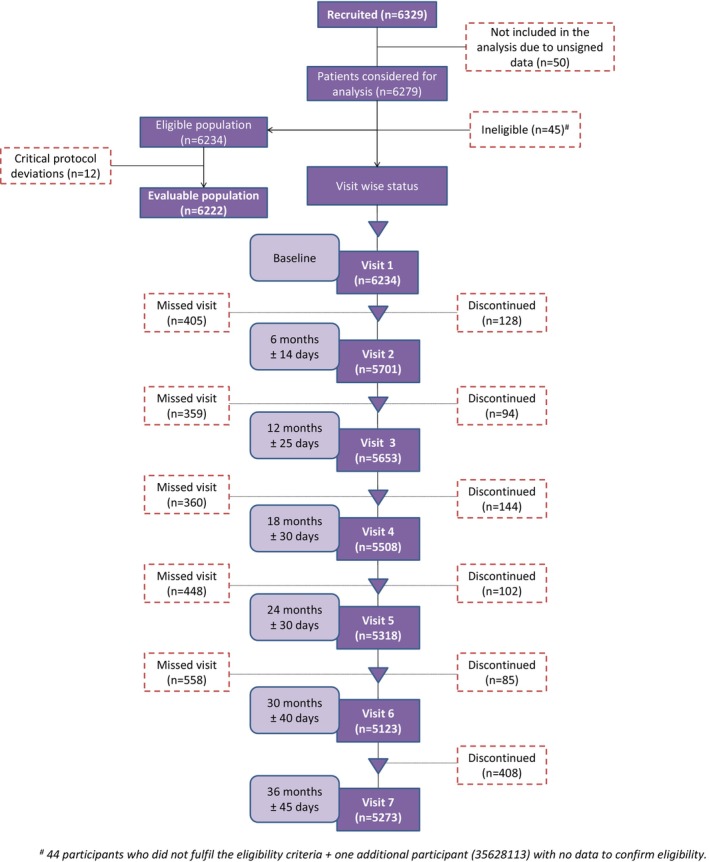
Participants disposition.

Of the 6279 participants recruited, 6234 fulfilled eligibility criteria and enrolled in the study. A total of 12 participants of this eligible population who had critical protocol deviation were excluded from the evaluable population (*N* = 6222). Overall, 5273 participants completed 3 years in this study, and 961 participants discontinued the study (Figure 1); the reasons for discontinuation are summarized in Table S1. The key reasons for discontinuation were participants lost to follow‐up (41.0%), site withdrawal from the study (17.6%) and withdrawal due to the pandemic (14.5%). There were a total of 53 deaths (5.5%), out of which 37 (3.9%) were cardiovascular deaths, and the remaining 16 deaths were due to unknown causes. Details of demographics and baseline characteristics are presented in Table S2. At baseline, the mean (SD) age of the participants was 52.1 (9.2) years with more than half (57.0%) of the population belonging to the age group 50–65 years and more than half of the participants being men (56.6%). The mean (SD) BMI was 27.2 (4.6) kg/m^2^, and majority of participants were obese (66.8%). At baseline, the median (range) duration of diabetes was 7.1 (4.3–11.1) years, and one‐third of participants were insulin naïve (75.2%).

### 3.2 Complications

#### 3.2.1 Macrovascular complications over 36‐months follow‐up

At the end of 3‐years, a total of 205 (3.3%) participants reported 216 macrovascular complications (including 148 complications existing at baseline and 68 new complications over 3 years of follow‐up). Nonfatal MI was the most common cardiovascular complication observed in 82 (40.0%) participants followed by PVD in 63 (30.7%), cardiovascular deaths in 37 (18.1%) and nonfatal stroke in 32 (15.6%) participants (Table 1). Among 37 cardiovascular deaths, the majority were sudden deaths (*n* = 24), followed by fatal MI (*n* = 10), coronary artery procedure (*n* = 2) and stroke (*n* = 1).

**TABLE 1:** Proportion of study participants with macrovascular disease over 36 months (*N* = 6222).

	Baseline	36 months (*n* = 6222)
Total number of macrovascular complications	148	216
Total number of people with macrovascular complications	144 (2.3)	205 (3.3)
New complications over 36 months	‐	68
Nonfatal myocardial infarction	74 (51.4)	82 (40.0)
Nonfatal stroke	29 (20.1)	32 (15.6)
Cardiovascular death	0	37 (18.1)
Peripheral vascular disease	45 (31.3)	63 (30.7)
No complications	5935	‐
Unknown	143	‐

*Note*: Data shown as *n* (%).

#### 3.2.2 Composite of nonfatal MI/nonfatal stroke/hospitalization for unstable angina/cardiovascular death over 36 months

The population showed an increasing trend over the visits in the incidence of these complications, from 1.6% at baseline, to 1.9% at 12‐months, to 2.2% at 24‐months. The cumulative incidence at the end of 36 months was 2.3% (Table 2).

**TABLE 2:** Summary of cumulative incidence of composite events (nonfatal MI/nonfatal stroke/hospitalization for unstable angina/cardiovascular death) over 36 months.

	People with composite events [m]	Number of new participants [m]
At baseline	101 (1.6) [103]	
At 6 months	113 (1.8) [116]	13 (0.2) [13]
At 12 months	120 (1.9) [125]	9 (0.1) [9]
At 18 months	125 (2.0) [132]	7 (0.1) [7]
At 24 months	137 (2.2) [144]	12 (0.2) [12]
At 30 months	142 (2.3) [149]	5 (0.1) [5]
At 36 months	146 (2.3) [154]	5 (0.1) [5]

*Note*: Data shown as n (%) [m]. m, number of events. Percentages are based on number of participants in evaluable population. People with at least one occurrence of nonfatal myocardial infarction or nonfatal stroke or hospitalization for unstable angina or cardiovascular death will be considered as ‘People with Composite event’.

#### 3.2.3 Participants with other cardiovascular events (hospitalization due to ACS, urgent revascularization procedures, HF or unstable angina) at the end of 6‐, 12‐, 24‐ and 36‐months

A total of five (0.08%) participants required urgent revascularization procedures during the study period (one participant at the end of 12‐months and four participants by the end of 24‐months). During the study period, eight (0.1%) participants required hospitalization for cardiovascular events (eight events); five of these participants were hospitalized for ACS (one, three and one at 6, 24 and 36 months, respectively), two for unstable angina (at 12 and 24 months) and one for HF (at 24 months) (Table S3).

#### 3.2.4 Microvascular complications at 12‐, 24‐ and 36‐months follow‐up

At the end of 36 months, a total of 1121 (18.0%) participants reported 1313 microvascular complications. Among these, the most frequently reported microvascular complication was neuropathy in 919 (82.0%) participants, while nephropathy and retinopathy were reported in 229 (20.4%) and 165 (14.7%) participants, respectively. A total of 283 new complications were reported during the post‐baseline follow‐up visits, and neuropathy accounted for around >60% of new microvascular complications reported at each follow‐up visit (Table 3).

**TABLE 3:** Development of microvascular complications at 12‐, 24‐ and 36‐months.

	Baseline	12 months	24 months	36 months	Total
Total number of microvascular complications	1030	117	129	37	1313
Total number of people with complications	900 (14.5)	114	124	35	1121 (18.0)
Neuropathy	735 (81.7)	80	79	25	919 (82.0)
Nephropathy	154 (17.1)	26	41	8	229 (20.4)
Retinopathy	141 (15.7)	11	9	4	165 (14.7)

*Note*: Data shown as *n* (%).

#### 3.2.5 Composite renal and retinal microvascular outcome

The cumulative incidence of renal and retinal complications showed an increasing trend over the follow‐up visits from 2.5% at baseline to 3.1% at 12‐months follow‐up, which further increased to 3.9% at 24‐months follow‐up and to 4.1% at the end of 36‐months follow‐up (Table 4).

**TABLE 4:** Summary of composite renal and retinal microvascular outcome.

	People with composite outcome (*N* = 6222)	Number of new participants
At baseline	154 (2.5)	‐
At 6 months	177 (2.8)	23 (0.4)
At 12 months	191 (3.1)	14 (0.2)
At 18 months	219 (3.5)	28 (0.5)
At 24 months	241 (3.9)	22 (0.4)
At 30 months	251 (4.0)	10 (0.2)
At 36 months	252 (4.1)	1 (0.02)

*Note*: Data shown as *n* (%). Percentages are based on number of participants in evaluable population. Composite outcome is the first occurrence of any of the following: Initiation of retinal photocoagulation, vitreous haemorrhage, diabetes‐related blindness or incident or worsening nephropathy.

### 3.3 Glycemic control

#### 3.3.1 Participants with controlled disease (HbA1c <7% and <6.5%) at the end of 6‐, 12‐, 24‐ and 36‐months

At the end of 36 months, 36.6% of participants had HbA1c <7% and 14.5% of the participants had HbA1c <6.5%, respectively. At baseline, 1119 (25.1%) participants had HbA1c <7% and 526 (11.8%) participants had HbA1c <6.5%. At the end of 6, 12 and 24 months, 28.9%, 29.6% and 33.4% of the participants had HbA1c <7% and 12.6%, 12.5% and 13.5% of the participants had HbA1c <6.5%, respectively. There was an increasing trend observed in the proportion of participants achieving glycemic control from baseline to the 36 months (Table 5).

**TABLE 5:** Proportion of participants who are controlled (HbA1c <7% and <6.5%) at the end of 6, 12, 24 and 36 months.

Visits	HbA1c <6.5%	HbA1c ≥6.5%	HbA1c <7%	HbA1c ≥7%
Baseline (*N* = 4466)	526 (11.8)	3940 (88.2)	1119 (25.1)	3347 (74.9)
6 months (*N* = 4082)	516 (12.6)	3566 (87.4)	1178 (28.9)	2904 (71.1)
12 months (*N* = 4225)	527 (12.5)	3698 (87.5)	1250 (29.6)	2975 (70.4)
24 months (*N* = 3879)	522 (13.5)	3357 (86.5)	1295 (33.4)	2584 (66.6)
36 months (*N* = 3700)	537 (14.5)	3163 (85.5)	1356 (36.6)	2344 (63.4)

*Note*: Data shown as *n* (%). HbA1c results are categorized based on reference glycemic targets: Standards of Medical Care in Diabetes‐2018, American Diabetes Association, Diabetes Care January 2018, 41 (Supplement 1) S55.

#### 3.3.2 Occurrence of microvascular and macrovascular complications in participants with controlled (HbA1c <7%) and uncontrolled (HbA1c ≥7%) disease at the end of 36 months

Among the 3696 participants who had HbA1c recorded at baseline as well as 36 months, a total of 101 participants had macrovascular complications and 659 had microvascular complications until the end of 36‐months. The participants who developed macrovascular or microvascular complications had a higher proportion of uncontrolled diabetes, 78.2% and 70.3%, respectively. In comparison, the participants who developed no complications until the end of the study had a lower proportion of uncontrolled diabetes (61.6%).

#### 3.3.3 Comparison of change in glycemic assessments at 36 months between participants with and without complications at baseline

**FIGURE 2:**
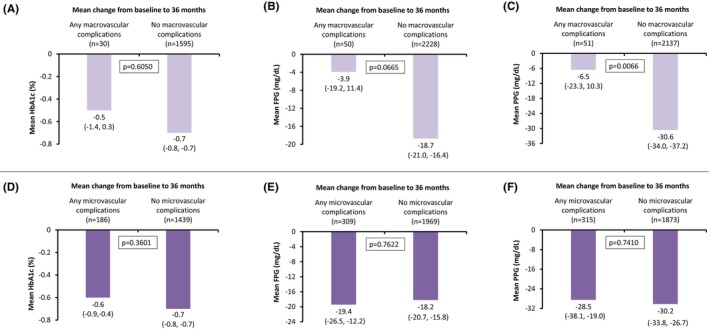
Comparison of change in glycemic assessments at 36 months between participants with and without complication at baseline. CI, confidence interval; HbA1c, glycated haemoglobin; FPG, fasting plasma glucose; PPG, postprandial plasma glucose.

A reduction in the mean change from baseline to 36‐months follow‐up was observed in all three glycemic parameters (HbA1c, FPG and PPG), for participants with and without macrovascular complications at baseline. However, only the reduction in the mean change for PPG levels was significantly lower in participants without macrovascular complications as compared with those with macrovascular complications at baseline (−30.6 mg/dL vs. −6.5 mg/dL; *p* = .0066) (Figure 2A–C). The mean changes from baseline to 36‐months follow‐up in all the three glycemic indices (HbA1c, FPG and PPG) between the participants with and without microvascular complications at baseline were comparable (Figure 2D–F).

#### 3.3.4 Comparison of change in glycemic control and outcomes at 36 months between participants with and without complications at baseline

**FIGURE 3:**
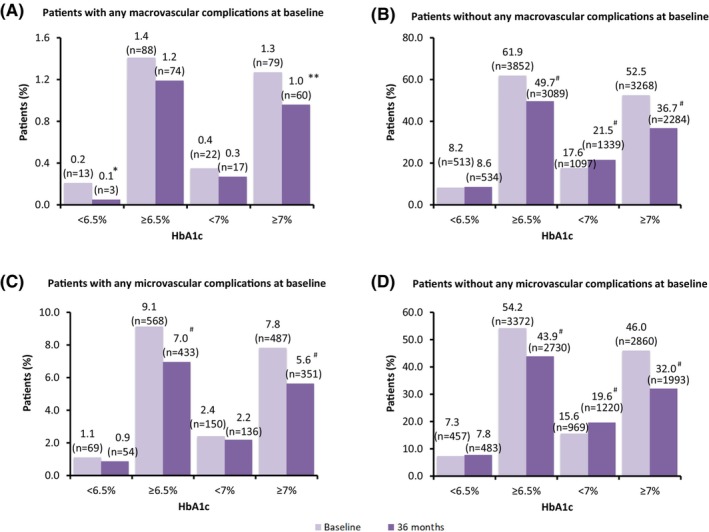
Comparison of change in glycemic control at 36 months between participants with and without complications at baseline. Percentages are based on number of study participants in evaluable population (*n* = 6222). The data presented as percentage (number of people). *p = .0039, **p = .0241, #*p* < .0001. HbA1c, glycated haemoglobin.

Among participants with macrovascular complications at baseline, there was a nonsignificant decrease in the proportion of participants reaching HbA1c <7% (0.4%–0.3%) (Figure 3A) while among participants without macrovascular complications at baseline, there was a significant increase in the proportion of participants reaching HbA1c <7% at the end of 36 months (17.6%–21.5%; *p* < .0001) (Figure 3B).

Among participants with microvascular complications at baseline, there was no significant difference in proportion of participants reaching HbA1c <7% at the end of 36 months (2.4%–2.2%) (Figure 3C) while among participants without microvascular complications at baseline, there was a significant increase in the proportion of participants reaching HbA1c <7% at the end of 36 months (from 15.6% to 19.6%; *p* < .0001) (Figure 3D).

Improvement in glycemic parameters in the insulin subgroup was significantly better compared with the insulin naïve subgroup (*p* < .0001) (Table S4).

### 3.4 Management (Time to treatment adaptation from baseline to 12‐, 24‐ and 36‐months)

#### 3.4.1 Shift in oral and injectable antidiabetic drugs categories

A majority (67.7%–73.9%) of the participants were taking only OADs as treatment from baseline to 36 months follow‐up. Addition of injectables (mostly insulin) was preferred in participants who were only on OADs at baseline, and switching of insulin types was done for participants on insulin treatment at baseline. The participants who were already on a combination of OADs and injectables had very minimal shifts in their treatment and tended to continue with their ongoing combination of treatment. In injectables antidiabetic drugs, half of the participants (50.7%) continued with basal insulin while 37.8% and 14.0% of participants continued with premix insulin and prandial insulin, respectively, over 36 months treatment period.

#### 3.4.2 Summary of OAD count by treatment

At baseline, around 95.7% of the evaluable population was taking at least 2 OADs and about 49.3% of participants were taking ≥3 OADs. About 3.9% of the participants were taking ≥5 OADs. Use of only 1 OAD was more common in participants on insulin (15.2%) than those in the insulin naïve group (0.2%). Correspondingly, nearly 99.7% of people who were insulin‐naïve at baseline were taking at least 2 OADs (Table 6).

**TABLE 6:** Summary of oral antidiabetic drug count at baseline, 12, 24 and 36 months.

Visits			Antidiabetic drug count				
		1 OAD	2 OADs	3 OADs	4 OADs	5 OADs	>5 OADs
Baseline [*N* = 6196]		244 (3.9)	2887 (46.4)	2011 (32.3)	814 (13.1)	185 (3.0)	55 (0.9)
	Insulin [*n* = 1541]	234 (15.2)	567 (36.8)	464 (30.1)	194 (12.6)	48 (3.1)	8 (0.5)
	Insulin naïve [*n* = 4681]	10 (0.2)	2320 (49.6)	1547 (33.0)	620 (13.2)	137 (2.9)	47 (1.0)
12 months [*N* = 5971]		222 (3.6)	1928 (31.0)	2010 (32.3)	1228 (19.7)	425 (6.8)	158 (2.5)
	Insulin [*n* = 1541]	172 (11.2)	443 (28.7)	440 (28.6)	272 (17.7)	91 (5.9)	22 (1.4)
	Insulin naïve [*n* = 4681]	50 (1.1)	1485 (31.7)	1570 (33.5)	956 (20.4)	334 (7.1)	136 (2.9)
24 months [*N* = 5716]		249 (4.0)	1674 (26.9)	1902 (30.6)	1208 (19.4)	454 (7.3)	229 (3.7)
	Insulin [*n* = 1541]	160 (10.4)	415 (26.9)	401 (26.1)	259 (16.8)	95 (6.2)	40 (2.6)
	Insulin naïve [*n* = 4681]	89 (1.9)	1259 (26.9)	1500 (32.0)	949 (20.3)	359 (7.7)	189 (4.0)
36 months [*N* = 5225]		234 (3.8)	1458 (23.4)	1739 (27.9)	1170 (18.8)	427 (6.9)	197 (3.2)
	Insulin [*n* = 1541]	148 (9.6)	355 (23.0)	374 (24.3)	248 (16.1)	89 (5.8)	34 (2.2)
	Insulin naïve [*n* = 4681]	86 (1.8)	1103 (23.6)	1365 (29.2)	922 (19.7)	338 (7.2)	163 (3.5)

*Note*: Data shown as *n* (%). Percentages are based on number of study participants using at least one OAD within each subgroup in evaluable population.

*Abbreviation*: OAD, oral antidiabetic drug.

Over the course of the study, the proportion of participants taking ≥3 OADs increased from 49.3% at baseline to 61.3% at 12‐months follow‐up. The proportion of participants taking ≥3 OADs remained at 61.0% at 24‐months and 56.8% at 36‐months follow‐up (Table 6).

#### 3.4.3 Shift in oral and injectable antidiabetic drugs

The proportion of participants taking injectable glucose‐lowering medications increased from 25.5% at baseline to 36.7% at 3 years, while the number of participants taking OADs remained stable (99.6% at baseline to 99.3% at 3 years).

**FIGURE 4:**
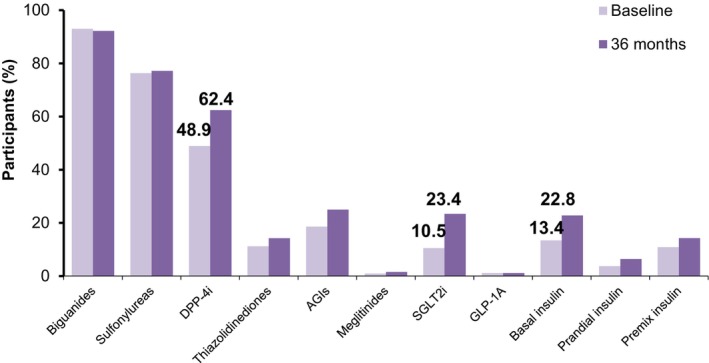
Comparison of the use of oral and injectable glucose‐lowering drugs at baseline and 36‐months. AGIs, alpha‐glucosidase inhibitors; DPP‐4i, dipeptidyl peptidase‐4 inhibitors; GLP‐1A, glucagon‐like peptide 1 analogue; SGLT2i, sodium‐glucose cotransporter 2 inhibitors.

At 36 months, biguanides (92.2%), sulfonylureas (77.28%) and DPP‐IV inhibitors (62.4%) were the commonly prescribed OADs and among injectable antidiabetic therapies, use of basal insulin (22.8%) and premix insulin (14.3%) was more common. The highest increase in OAD addition from baseline to 36 months was seen for DPP‐IV inhibitors (48.9%–62.4%), followed by sodium‐glucose cotransporter 2 (SGLT2) inhibitors (10.5%–23.4%) (Figure 4).

Most of the participants taking OADs were being treated with biguanides (90.8%–93.4%), sulfonylureas (72.1%–77.0%) and DPP‐IV inhibitors (44.9%–49.1%) across the follow‐up visits. There was a slight increase (from 40.2% participants at baseline to 46.5% participants at 36 months) in the participants taking DPP‐IV inhibitors among those who were on sulfonylureas at baseline.

In injectables, the majority of participants continued to receive the same type of insulin as at baseline. About one in five participants on basal had prandial insulin co‐administered. There was also a shift in the proportion of participants taking GLP‐1 analogues at baseline towards basal, premix or prandial insulin.

#### 3.4.4 Change in dose of OADs categories

At 12‐, 24‐ and 36‐months visit, dose modifications such as increase/decrease in dose, addition or discontinuation of an OAD occurred mostly in <0.5% of the participants in each drug count category. Most of the dose modifications were done at around 6 months (Table S5).

#### 3.4.5 Change in dose of insulin categories

The mean (SD) change from baseline in total daily dose of basal insulin indicated an increase in dose by 1.6 (7.8) U at 12‐months; 1.7 (10.4) U at 24‐months and 1.9 (9.8) U at 36‐months. For prandial insulin, the mean (SD) change from baseline in total daily dose indicated an increase in dose by 2.2 (11.3) U at 12‐months; 2.8 (19.2) U at 24‐months and 3.3 (20.0) U at 36‐months. The mean (SD) change from baseline in total daily dose indicated an increase in premix insulin dose by 2.3 (10.4) U at 12‐months, and 2.9 (16.4) U at 36‐months (Table S6).

### 3.5 Hypoglycaemic events, hospitalization and adverse drug reactions

During the 3‐year study period, 91 hypoglycaemic events were reported. The majority of the cases were documented symptomatic hypoglycaemia (no. of events [m] = 45), followed by asymptomatic hypoglycaemia (m = 23), nocturnal hypoglycaemia (m = 17) and six events of severe hypoglycaemia events. A total of five (0.08%) participants required hospitalization due to nonfatal MI during the study period (two participants at 6 months, one participant at 24 months and two participants at the end of 36 months). One (0.02%) participant required hospitalization due to a nonfatal stroke by the end of 6 months. There were no serious ADRs, fatal ADRs or ADRs suspected of transmission of an infectious agent via medicinal product reported during 3 years of follow‐up visits.

## 4 DISCUSSION

Type 2 diabetes in the Indian population is recognized to have a few distinctive clinical features such as high incidence and prevalence, disease occurrence at a young age and low BMI, high proportion of individuals affected with coronary artery disease, low prevalence of PVD and probable low incidence of microvascular complications.^1^ Poor glycemic control is also a primary observation in the Indian population and suggests a lack of optimal diabetes management strategies in routine clinical practice.^3^, ^5^ The real‐world data was gathered during a 3‐years follow‐up period in the LANDMARC study, which enrolled a sizable pan‐Indian diabetes population (6279 participants with a mean age of 52.1 years and a median duration of diabetes of 7.1 years, two‐fifths with coexisting hypertension and one‐third with dyslipidemia at enrollment), has provided crucial insights into the complications, glycemic control and management of diabetes.

During the 36‐month follow‐up period, the prevalence of macrovascular complications increased from 2.3% to 3.3% among study participants. Our findings, which focused solely on the hard end‐points, are similar to a multicountry 3‐year prospective observational study (DISCOVER) that reported a crude prevalence of macrovascular complications of 4% in South East Asia subgroup (India and Indonesia, *n* = 3360) using a much broader definition of macrovascular complications.^14^ Nonfatal MI followed by PVD were the commonly reported macrovascular complications in the present study. The DISCOVER program also reported coronary artery disease (MI) as the most commonly observed macrovascular disease among people with T2DM.^14^ The prevalence of PVD in the T2DM population reported in the literature from India ranges between 8.6% and 15.1%, while our real‐world observational study showed relatively higher rates (30.7%),^15^, ^16^ highlighting the need to screen individuals for PVD regularly. At the end of 3‐years, there was an increase in the cumulative incidence of composite of nonfatal MI/nonfatal stroke/hospitalization for unstable angina/cardiovascular death (2.3% from 1.6%) from baseline in the present study population. Cardiovascular event was the leading cause of death in the study population.

According to the present study, neuropathy was the most common complication among the 18% of participants who had microvascular complications. Similar observations have been reported by previous Indian as well global studies like DISCOVER study (prevalence ranging between 7.7% and 60.4%).^14^, ^15^, ^17^, ^18^, ^19^, ^20^ In comparison to the current real‐world observations, the TIGHT study found a higher prevalence of microvascular complications (35.7%), with neuropathy being the most common.^4^ In a prior LANDMARC interim analysis at 2 years, it was observed that microvascular complications were significantly higher in participants from nonmetropolitan than metropolitan cities (12.9% vs. 4.7%; *p* < .0001), which also may need further exploration.^8^ Furthermore, the 2‐year data from LANDMARC indicated more complications among participants with CV risk factors, higher BMI or suboptimal glycemic control.^11^ Similarly, the additional analysis of 3‐year data from LANDMARC indicates more complications among those with suboptimal glycemic control (*p* < .0001) or those having CV risk factors (*p* < .0001) (Data not presented).

At the end of 3‐years, there was an increase in the cumulative incidence of composite renal and retinal microvascular outcomes (4.1% from 2.5%) from baseline in the present study population. People with microvascular alterations in the retina are more prone to the risk of developing diabetic kidney disease. Therefore, screening of people with uncontrolled T2DM for retinal microvascular signs may help in the early diagnosis of people at‐risk of end‐stage renal disease.^21^ An increase in composite end‐points over the 3‐years of study period might suggest the trend of progression of diabetes and associated complications despite Indian people being on current antidiabetic therapies. Therefore, there is a need of optimizing strategies with timely intensification for diabetes management in India.

Poor glycemic control is strongly associated with the risk of development of microvascular and macrovascular complications. A substantial body of evidence supports the role of aggressive glycemic control in lowering the risk of developing or progressing to these complications.^17^, ^22^, ^23^, ^24^ In the present study, at the end of the 3‐year follow‐up, the population with macrovascular and microvascular complications had a higher proportion of participants with uncontrolled glycemia compared with those without complications. This result emphasizes the importance of persistent achievement of target blood glucose levels to reduce the risk of macrovascular or microvascular complications. Individuals with young‐onset diabetes have a longer disease course and are more prone to develop severe complications.^25^ Therefore, vigilant monitoring to achieve good control over blood glucose levels and cardiovascular risk factors for a lifetime are vital,^25^ especially in India.

In terms of glycemic control, the decrease in the proportion of people with uncontrolled diabetes in this real‐world study showed a trend towards improvement in glycemic control over 3‐years of study duration. Although there was good improvement in all the glycemic parameters from baseline, a large majority of the participants remained uncontrolled in achieving target blood glucose at the end of 3 years.

The gradual trend of improvement in the glycemic control observed over 3 years in the present real‐world study is around 36% and is similar to that reported in the previous literature from India, wherein the proportion of people achieving glycemic control ranges between 23.4% and 31.0%.^1^, ^26^ This comprehensively portrays the current real‐life burden of uncontrolled diabetes among the Indian population. Reports from a real‐world study from India (the TIGHT study) are in accordance with this observation, wherein a high burden (76.6%) of poor glycemic control was recorded.^3^, ^4^, ^26^, ^27^

According to global as well as national guidelines, achieving and maintaining optimal glycemic targets over a long duration is the fundamental goal of the management of diabetes.^23^, ^28^ In addition, a patient‐centric approach is also important, wherein all the metabolic factors are considered while the treatment decision is being taken. These include cardiovascular risks, weight management, improvements in quality of life, patient preference and treatment affordability.^23^ The present study findings also highlight the therapy patterns among participants with T2DM across Indian healthcare settings. Almost all participants were on OADs with biguanides, sulfonylureas and DPP‐IV inhibitors being the most commonly used OADs. There was a gradual increase in insulin use from 25.5% at baseline to 36.7% at the end of 3 years. These treatment strategies are in line with the various guidelines wherein initiation of insulin is recommended when glycemic targets are not achieved with OADs.^23^, ^28^ In the current study, the insulin subgroup had significantly (*p* < .001) better glycemic control than the insulin‐naive participants. Increased use of insulin may be one of the attributable factors for the improvement in glycemic control observed over 3 years in this study. In the present study, treatment intensification occurred in very few participants and mostly within the first year of follow‐up. The addition of insulin for those who were only on OADs seemed a preference in treatment modification. In this real‐world study, a relatively lower proportion of the study population achieved glycemic control, with around half of the population on ≥3 OADs at the end of 3‐years, highlighting the need for early initiation of insulin therapy for achieving optimal glycemic control among Indian people with uncontrolled T2DM. The prescription pattern reported in a study by Singla R, et al. is similar to that observed in this study. Metformin was commonly prescribed for people having T2DM for 0–5 years duration, whereas sulfonylurea and DPP‐4 inhibitors were commonly prescribed for people with a disease duration of more than 5 years.^29^ In addition, the therapy trend observed in another cross‐sectional study from India was in concordance with the LANDMARC study.^30^

The key strength of this study is the longitudinal design, the pan‐ India representation in adequate numbers and the completion of the study by an acceptable proportion of participants. Although this is a pivotal real‐world study that represents the largest population of individuals with diabetes and their management across pan‐India locations, there are several limitations that cannot be ignored. Firstly, missing values during the 3‐years follow‐up may hamper the actual depiction of real‐world clinical experience. Secondly, we could not discern the impact of socioeconomic status and lifestyle factors (smoking and alcohol consumption) on overall diabetes management. Lastly, lack of data on cost‐effectiveness of different treatment approaches and treatment adherence made it difficult to understand the reasons behind uncontrolled glycemia in the majority of Indian people with diabetes.

## 5 CONCLUSION

The observations from this pivotal real‐world study provide valuable insights into diabetes management (in terms of diabetes‐related complications, disease progression and treatment strategies) in the Indian population and emphasize the need for optimizing diabetes management in India. This pan‐India study highlights the challenges faced due to the burden of uncontrolled glycemia and cumulative diabetes‐related complications in the management of T2DM in the Indian population. Nonfatal MI and neuropathy were the most prevalent macrovascular and microvascular complications, respectively, with death due to cardiovascular events being the leading cause of mortality in this population. Oral antidiabetic agents continue to remain the mainstay of T2DM treatment despite a large proportion of participants having uncontrolled glycemia. Timely intensification of therapy, including initiation of insulin, can help improve glycemic parameters. Development of macrovascular and microvascular complications can be prolonged/delayed if target glycemic control is achieved and sustained for a long period using appropriate treatment intensifications in people with diabetes in India.

## AUTHOR CONTRIBUTIONS

**Ashok Kumar Das:** Conceptualization (equal); data curation (supporting); formal analysis (supporting); funding acquisition (supporting); investigation (lead); methodology (equal); project administration (supporting); resources (supporting); software (supporting); supervision (lead); validation (lead); visualization (lead); writing – original draft (equal); writing – review and editing (equal). **Sanjay Kalra:** Conceptualization (supporting); data curation (supporting); formal analysis (supporting); funding acquisition (supporting); investigation (equal); methodology (supporting); project administration (supporting); resources (supporting); software (supporting); supervision (lead); validation (lead); visualization (lead); writing – original draft (equal); writing – review and editing (equal). **Shashank R Joshi:** Conceptualization (supporting); data curation (supporting); formal analysis (supporting); funding acquisition (supporting); investigation (equal); methodology (supporting); project administration (supporting); resources (supporting); software (supporting); supervision (equal); validation (equal); visualization (equal); writing – original draft (equal); writing – review and editing (equal). **Ambrish Mithal:** Conceptualization (supporting); data curation (supporting); formal analysis (supporting); funding acquisition (supporting); investigation (equal); methodology (supporting); project administration (supporting); resources (supporting); software (supporting); supervision (equal); validation (equal); visualization (equal); writing – original draft (equal); writing – review and editing (equal). **KM Prasanna Kumar:** Conceptualization (supporting); data curation (supporting); formal analysis (supporting); funding acquisition (supporting); investigation (equal); methodology (supporting); project administration (supporting); resources (supporting); software (supporting); supervision (equal); validation (equal); visualization (equal); writing – original draft (equal); writing – review and editing (equal). **AG Unnikrishnan:** Conceptualization (supporting); data curation (supporting); formal analysis (supporting); funding acquisition (supporting); investigation (equal); methodology (supporting); project administration (supporting); resources (supporting); software (supporting); supervision (equal); validation (equal); visualization (equal); writing – original draft (equal); writing – review and editing (equal). **Hemant Thacker:** Conceptualization (supporting); data curation (supporting); formal analysis (supporting); funding acquisition (supporting); investigation (equal); methodology (supporting); project administration (supporting); resources (supporting); software (supporting); supervision (equal); validation (equal); visualization (equal); writing – original draft (equal); writing – review and editing (equal). **Bipin Sethi:** Conceptualization (supporting); data curation (supporting); formal analysis (supporting); funding acquisition (supporting); investigation (equal); methodology (supporting); project administration (supporting); resources (supporting); software (supporting); supervision (equal); validation (equal); visualization (equal); writing – original draft (equal); writing – review and editing (equal). **Subhankar Chowdhury:** Conceptualization (supporting); data curation (supporting); formal analysis (supporting); funding acquisition (supporting); investigation (equal); methodology (supporting); project administration (supporting); resources (supporting); software (supporting); supervision (equal); validation (equal); visualization (equal); writing – original draft (equal); writing – review and editing (equal). **Amarnath Sugumaran:** Conceptualization (equal); data curation (equal); formal analysis (supporting); funding acquisition (equal); investigation (equal); methodology (equal); project administration (equal); resources (supporting); software (supporting); supervision (equal); validation (equal); visualization (equal); writing – original draft (equal); writing – review and editing (equal). **Ashwini Satpathy:** Conceptualization (equal); data curation (equal); formal analysis (supporting); funding acquisition (equal); investigation (equal); methodology (equal); project administration (equal); resources (supporting); software (supporting); supervision (equal); validation (equal); visualization (equal); writing – original draft (equal); writing – review and editing (equal). **Arvind Gadekar:** Conceptualization (equal); data curation (equal); formal analysis (supporting); funding acquisition (equal); investigation (equal); methodology (equal); project administration (equal); resources (supporting); software (supporting); supervision (equal); validation (equal); visualization (equal); writing – original draft (equal); writing – review and editing (equal). **Shalini Kesav Menon:** Conceptualization (equal); data curation (equal); formal analysis (supporting); funding acquisition (equal); investigation (equal); methodology (equal); project administration (equal); resources (supporting); software (supporting); supervision (equal); validation (equal); visualization (equal); writing – original draft (equal); writing – review and editing (equal). **Renuka Neogi:** Conceptualization (supporting); data curation (equal); formal analysis (equal); funding acquisition (supporting); investigation (equal); methodology (equal); project administration (equal); resources (supporting); software (equal); supervision (equal); validation (equal); visualization (equal); writing – original draft (equal); writing – review and editing (equal). **Deepa Chodankar:** Conceptualization (equal); data curation (equal); formal analysis (lead); funding acquisition (supporting); investigation (equal); methodology (lead); project administration (equal); resources (lead); software (lead); supervision (equal); validation (equal); visualization (equal); writing – original draft (equal); writing – review and editing (equal). **Chirag Trivedi:** Conceptualization (equal); data curation (equal); formal analysis (equal); funding acquisition (equal); investigation (equal); methodology (equal); project administration (equal); resources (equal); software (supporting); supervision (equal); validation (equal); visualization (equal); writing – original draft (equal); writing – review and editing (equal). **Subhash Kumar Wangnoo:** Conceptualization (supporting); data curation (supporting); formal analysis (supporting); funding acquisition (supporting); investigation (equal); methodology (supporting); project administration (supporting); resources (supporting); software (supporting); supervision (equal); validation (equal); visualization (equal); writing – original draft (equal); writing – review and editing (equal). **Abdul Zargar:** Conceptualization (supporting); data curation (supporting); formal analysis (supporting); funding acquisition (supporting); investigation (equal); methodology (supporting); project administration (supporting); resources (supporting); software (supporting); supervision (equal); validation (equal); visualization (equal); writing – original draft (equal); writing – review and editing (equal). **Nadeem Rais:** Conceptualization (supporting); data curation (supporting); formal analysis (supporting); funding acquisition (supporting); investigation (equal); methodology (supporting); project administration (supporting); resources (supporting); software (supporting); supervision (equal); validation (equal); visualization (equal); writing – original draft (equal); writing – review and editing (equal).

## FUNDING INFORMATION

This study was funded by Sanofi.

## CONFLICT OF INTEREST STATEMENT

AKD, AM, AGU and NR received honoraria from Sanofi and other pharmaceutical companies. KMPK is on the advisory board of Sanofi and received honorarium for his talks. SJ received speaker/advisory/research grants from Abbott, AstraZeneca, Biocon, Boehringer Ingelheim (BI), Eli Lilly, Franco Indian, Glenmark, Lupin, Marico, MSD, Novartis, Novo Nordisk, Roche, Sanofi, Serdia, Twinhealth and Zydus. SK received honoraria/speaker fees from Eli Lilly, Novo Nordisk and Sanofi. HT received honoraria from MSD, Novartis, Sanofi and from other companies for advice and lectures. BS received honorarium from Aventis, Novo Nordisk, Eli Lilly, BI and MSD. SC received honoraria/grants from Biocon, BI, Intas, Novartis, Sanofi and Serdia. SKW has nothing to declare. AHZ received honoraria from Novo Nordisk, Eli Lilly, Johnson & Johnson, AstraZeneca, BI and Sanofi. SKM, AG, ASugumaran, ASatpathy, RN, DC and CT are/were employees of Sanofi and may hold stock options.

## Supporting information


Table S1.–S6.

## Data Availability

Qualified researchers may request access to person‐level data and related study documents including the clinical study report, study protocol with any amendments, blank case report form, statistical analysis plan and data set specifications. Person‐level data will be anonymized, and study documents will be redacted to protect the privacy of study participants. Further details on Sanofi's data sharing criteria, eligible studies and the process for requesting access can be found at https://vivli.org/.
