# Supplementary material for: The LongitudinAl Nationwide stuDy on Management And Real‐world outComes of diabetes in India over 3 years (LANDMARC trial)
Source: Endocrinol Diabetes Metab. 2023 Jul 1;6(5):e422. doi: 10.1002/edm2.422 (PMC10495555; doi:10.1002/edm2.422)
Supplement: Supplementary file 1 — Table S1.–S6. [file EDM2-6-e422-s001.docx]

**Supplementary Material**

**Supplementary table 1:** Reasons for discontinuation

| **Reasons** | **Total (Sites=382) (N=6234) n (%)** |
| --- | --- |
| Participants who discontinued from the study | 961 (15.4) |
| Death | 53 (5.5) |
| Death (Cardiovascular) | 37 (3.9) |
| Death (Other) | 16 (1.7) |
| Participant does not wish to continue | 54 (5.6) |
| Adverse Drug Reaction | 0 |
| Lost to follow-up | 394 (41.0) |
| Discontinued due to site withdrawal | 169 (17.6) |
| Other | 291 (30.3) |
| Pandemic | 139 (14.5) |
| Missed Visit | 81 (8.4) |
| Principal investigator withdrew | 28 (2.9) |
| Participant has shifted/migrated to another place | 25 (2.6) |
| Inability to identify subject for follow-up visit due to loss of all documents | 14 (1.5) |
| Developed Stroke | 1 (0.1) |
| Diabetic foot complication | 1 (0.1) |
| Participant couldn't visit due to severe renal problem | 1 (0.1) |
| Participant developed cancer and is on treatment in another hospital | 1 (0.1) |

**Supplementary table 2:** Baseline characteristics (Eligible population)

| **Parameters** | **Participants (N=6234)** |
| --- | --- |
| Age (years), mean (SD) | 52.1 (9.2) |
| Age group (years) |  |
| ≤30 | 61 (1.0) |
| 31-49 | 2192 (35.2) |
| 50-65 | 3552 (57.0) |
| ≥66 | 429 (6.9) |
| Gender, n (%) |  |
| Male | 3526 (56.6) |
| Female | 2708 (43.4) |
| BMI (N=6215), mean (SD) | 27.2 (4.6) |
| BMI group (kg/m^2^) |  |
| Underweight | 44 (0.7) |
| Normal | 903 (14.5) |
| Overweight | 1119 (18.0) |
| Obese | 4149 (66.8) |
| Duration of disease (years), median (range) | 7.1 (4.3-11.1) |
| Duration of disease (years)  2-6 | 2359 (37.8) |
| 6-10 | 2147 (34.4) |
| >10 | 1728 (27.7) |
| Cities |  |
| Metro | 2376 (38.1) |
| Non-metro | 3858 (61.9) |
| HbA1c (%), mean (SD) | 8.1 (1.6) |
| Glycemic status (N=4477) |  |
| < 7.0 % | 1121 (25.0) |
| ≥ 7.0 % | 3356 (75.0) |
| Treatment |  |
| Insulin | 1549 (24.8) |
| Insulin-naive | 4685 (75.2) |
| Cardiovascular risk factors | N=3282 |
| Hypertension | 2566 (78.2) |
| Dyslipidemia | 1636 (49.8) |
| Albuminuria | 153 (4.7) |
| Family history of PCD | 65 (2.0) |
| Data shown as n (%), unless otherwise specified.  Abbreviations: HbA1c, glycated hemoglobin; PCD, premature coronary disease; SD, standard deviation. | |

**Supplementary table 3:** Proportion of study participants with other cardiovascular events such as hospitalization due to ACS, urgent revascularization procedures, hospitalization for heart failure or unstable angina at the end of 6, 12, 24, and 36 months

|  | **At the end of** | | | |
| --- | --- | --- | --- | --- |
| **Events** | **6 months** | **12 months** | **24 months** | **36 months** |
| Total urgent Revascularization Procedures | - | 1 (0.02) [1] | 5 (0.08) [5] | 5 (0.08) [1] |
| New cases | - | 1 (0.02) [1] | 1 (0.02) [1] | - |
| Total hospitalization due to ACS | 1 (0.02) [1] | 1 (0.02) [1] | 4 (0.06) [4] | 5 (0.08) [5] |
| New cases | 1 (0.02) [1] | - | 1 (0.02) [1] | 1 (0.02) [1] |
| Total hospitalization due to heart failure | - | - | 1 (0.02) [1] | 1 (0.02) [1] |
| New cases | - | - | 1 (0.02) [1] | - |
| Total hospitalization due to unstable angina | - | 1 (0.02) [1] | 2 (0.03) [2] | 2 (0.03) [2] |
| New cases | - | 1 (0.02) [1] | 1 (0.02) [1] | - |
| Data shown as n (%) [number of events].  Abbreviation: ACS, acute coronary syndrome | | | | |

**Supplementary table 4:** Therapy trends at 36 months follow-up

|  |  | **Insulin naïve** | **Insulin** | **P value (between treatment subgroups)**** |
| --- | --- | --- | --- | --- |
| **A1C (%)** | n | 2279 | 823 | <0.001 |
|  | Mean | 7.2 | 7.6 |  |
|  | 95% CI | 7.1, 7.2 | 7.5, 7.7 |  |
| **Change in A1C (%) from baseline** | n | 2189 | 675 |  |
|  | Mean | -0.6 | -1.1 |  |
|  | 95% CI | -0.6, -0.5 | -1.2, -0.9 |  |
|  | P value* | <0.001 | <0.001 |  |
| **FPG (mg/dL)** | n | 2500 | 936 | <0.001 |
|  | Mean | 121.0 | 130.1 |  |
|  | 95% CI | 119.9, 122.0 | 127.7, 132.6 |  |
| **Change in FPG (mg/dL) from baseline** | n | 2595 | 833 |  |
|  | Mean | -14.0 | -24.4 |  |
|  | 95% CI | -16.0, -12.0 | -28.7, -20.0 |  |
|  | P value* | <0.001 | <0.001 |  |
| **PPG (mg/dL)** | n | 2393 | 916 | <0.001 |
|  | Mean | 171.2 | 186.0 |  |
|  | 95% CI | 169.6, 172.9 | 182.6, 189.5 |  |
| **Change in PPG (mg/dL) from baseline** | n | 2514 | 813 |  |
|  | Mean | -23.3 | -38.8 |  |
|  | 95% CI | -26.2, -20.3 | -45.0, -32.6 |  |
|  | P value* | <0.001 | <0.001 |  |
| *p-value is calculated for the change from the baseline using paired t-test. **p-value is calculated between the treatment subgroups using independent t-test.  Abbreviations: A1C, glycated hemoglobin; CI, confidence interval; FPG, fasting plasma glucose; PPG, post prandial glucose. | | | | |

**Supplementary table 5:** Change in dose of oral anti-diabetic drug categories

|  | **Visit 1 (Baseline)** | | | | | |
| --- | --- | --- | --- | --- | --- | --- |
| **Visit   Change in Dose** | **1 OAD (N=244)** | **2 OADs (N=2887)** | **3 OADs (N=2011)** | **4 OADs (N=814)** | **5 OADs (N=185)** | **>5 OADs (N=55)** |
| **6 months follow-up** |  |  |  |  |  |  |
| Increased dose | 7 (0.1) | 148 (0.1) | 98 (0.1) | 54 (0.1) | 17 (0.1) | 5 (0.1) |
| Decreased dose | 3 (0.1) | 56 (0.1) | 58 (0.1) | 42 (0.1) | 7 (0.1) | 2 (0.1) |
| Added new OAD | 45 (0.7) | 692 (0.7) | 375 (0.7) | 120 (0.7) | 27 (0.7) | 8 (0.7) |
| Stopped an OAD | 19 (0.3) | 172 (0.3) | 187 (0.3) | 90 (0.3) | 36 (0.3) | 15 (0.3) |
| **12 months follow-up** |  |  |  |  |  |  |
| Increased dose | 4 (0.1) | 105 (0.1) | 110 (0.1) | 52 (0.1) | 14 (0.1) | 4 (0.1) |
| Decreased dose | 4 (0.1) | 50 (0.1) | 66 (0.1) | 23 (0.1) | 5 (0.1) | 2 (0.1) |
| Added new OAD | 28 (0.5) | 392 (0.5) | 269 (0.5) | 110 (0.5) | 16 (0.5) | 6 (0.5) |
| Stopped an OAD | 13 (0.2) | 163 (0.2) | 177 (0.2) | 113 (0.2) | 30 (0.2) | 11 (0.2) |
| **24 month follow-up** |  |  |  |  |  |  |
| Increased dose | 4 (0.1) | 112 (0.1) | 97 (0.1) | 42 (0.1) | 10 (0.1) | 1 (0.1) |
| Decreased dose | 2 (0.03) | 55 (0.03) | 69 (0.03) | 35 (0.03) | 10 (0.03) | 1 (0.03) |
| Added new OAD | 14 (0.2) | 259 (0.2) | 201 (0.2) | 93 (0.2) | 22 (0.2) | 4 (0.2) |
| Stopped an OAD | 11 (0.2) | 152 (0.2) | 154 (0.2) | 105 (0.2) | 20 (0.2) | 9 (0.2) |
| **36 months follow-up** |  |  |  |  |  |  |
| Increased dose | 2 (0.03) | 48 (0.03) | 47 (0.03) | 12 (0.03) | 4 (0.03) | 2 (0.03) |
| Decreased dose | 0 (0.0) | 26 (0.0) | 27 (0.0) | 11 (0.0) | 1 (0.0) | 1 (0.0) |
| Added new OAD | 8 (0.1) | 145 (0.1) | 116 (0.1) | 46 (0.1) | 8 (0.1) | 2 (0.1) |
| Stopped an OAD | 4 (0.1) | 91 (0.1) | 82 (0.1) | 46 (0.1) | 13 (0.1) | 3 (0.1) |
| Percentages are based on evaluable Population. If study participant reported change in dose in more than one OAD, participant is counted once in all corresponding 'Change in Dose' categories. Abbreviation: OAD, oral anti-diabetic drug | | | | | | |

**Supplementary table 6:** Change in insulin dose

| **Visits** | **n** | **Mean (SD)** | **n** | **Mean change from baseline** |
| --- | --- | --- | --- | --- |
| **Basal insulin** | | | | |
| Baseline | 836 | 24.3 (18.9) | - | - |
| 12 months | 1129 | 24.3 (18.3) | 770 | 1.6 (7.8) |
| 24 months | 1187 | 23.9 (17.2) | 702 | 1.7 (10.4) |
| 36 months | 1199 | 23.5 (16.3) | 628 | 1.9 (9.8) |
| **Prandial insulin** | | | | |
| Baseline | 231 | 32.5 (21.4) | - | - |
| 12 months | 305 | 33.3 (24.8) | 208 | 2.2 (11.3) |
| 24 months | 325 | 33.5 (22.8) | 191 | 2.8 (19.2) |
| 36 months | 338 | 33.5 (22.3) | 174 | 3.3 (20.0) |
| **Premix insulin** | | | | |
| Baseline | 676 | 36.2 (22.9) | - | - |
| 12 months | 812 | 37.2 (24.9) | 616 | 2.3 (10.4) |
| 24 months | 842 | 36.6 (22.6) | 563 | 2.3 (14.4) |
| 36 months | 750 | 36.6 (23.1) | 468 | 2.9 (16.4) |
